# Role of routine abdominal ultrasonography in intensified tuberculosis case finding algorithms at HIV clinics in high TB burden settings

**DOI:** 10.1186/s12879-017-2433-6

**Published:** 2017-05-18

**Authors:** Sonam Spalgais, Upasna Agarwal, Rohit Sarin, Devesh Chauhan, Anita Yadav, Anand Jaiswal

**Affiliations:** 1 National Institute of TB and Respiratory Diseases, Sri Aurobindo Marg, New Delhi, 110030 India; 2 Kusuma School of Biological Sciences IIT Delhi, New Delhi, Hauz Khaz, 110016 India

**Keywords:** HIV, TB, TB/HIV, USG, Abdominal TB

## Abstract

**Background:**

High proportion of TB in people living with HIV (PLHIV) is undiagnosed. Due to this active TB case finding is recommended for HIV clinics in high TB burden countries. Presently sputum examination and chest radiography are frontline tests recommended for HIV infected TB presumptives. Abdominal TB which occurs frequently in PLHIV may be missed even by existing programmatic intensified case finding protocols. This study evaluated the routine use of ultrasonography (USG) for active case finding of abdominal TB in HIV clinics.

**Methods:**

Retrospective analysis of eight years’ data from an HIV Clinic in a TB hospital in India. Patients underwent chest x-ray, sputum examination, USG abdomen and routine blood tests at entry to HIV care. Case forms were scrutinized for diagnosis of TB, USG findings and CD4 cell counts. Abdominal TB was classified as probable or possible TB. Probable TB was based on presence of two major USG (abdomen) findings suggestive of active TB, or one major USG finding with at least two minor USG findings or at least two symptoms, or any USG finding with microbiologically confirmed active TB at another site. Possible TB was based on the presence of one major USG finding, or the presence of two minor USG findings with at least two symptoms. Bacteriological confirmation was not obtained.

**Results:**

Eight hundred and eighty-nine people PLHIV underwent a baseline USG abdomen**.** One hundred and thirteen of 340 cases already diagnosed with TB and 87 of the 91 newly diagnosed with TB at time of HIV clinic registration had abdominal TB. Non-abdominal symptoms like weight loss, fever and cough were seen in 53% and 22% cases had no symptoms at all. Enlarged abdominal lymph nodes with central caseation, ascitis, splenic microabsesses, bowel thickening and hepatosplenomegaly were the USG findings in these cases.

**Conclusions:**

Abdominal TB is a frequent TB site in PLHIV presenting with non-abdominal symptoms. It can be easily detected on basis of features seen on a simple abdominal ultrasound. Abdominal USG should be essential part of intensified TB case finding algorithms for HIV infected people living in high TB burden settings.

## Background

Human immunodeficiency virus (HIV) associated tuberculosis (TB) continues to be a problem for both TB and HIV programs. As per the 2015 World Health Organization (WHO) report, globally only 50% of the estimated new HIV-TB cases were actually reported. The remaining either went undiagnosed or if diagnosed, the quality of care for people in this category is unknown [1]. Difficulties in correct and timely diagnosis of active TB in HIV infected populations is a barrier to effective HIV care, contributing to ongoing high morbidity and mortality seen with HIV associated TB [1, 2]. For this reason, active TB case finding is recommended for HIV clinics in high TB burden countries. Presently sputum examination and chest radiographs are the recommended frontline tests for screening of HIV infected TB presumptives. However, not only is active TB in people living with HIV (PLHIV) often extra pulmonary, its diagnosis is challenging due to the atypical presentations of TB, the frequent involvement of sites which are difficult to access which makes obtaining a clinical specimen difficult (especially in low-resource settings) and the presence of multiple opportunistic infections with overlapping symptoms [3, 4].

Abdominal TB, though infrequent in HIV uninfected people, is a common site of active TB in people living with HIV, especially in high TB burden countries [5–7] and may either present as the only site of active TB or may be seen alongside other affected sites like pulmonary, pleural, peripheral and intra thoracic lymph nodes [8]. TB of the abdomen may have non-specific symptoms and clinical specimens for bacteriological confirmation may not be easily available [4, 8]. This makes its diagnosis difficult and the disease may go undetected unless specifically looked for. The morbidity and mortality of HIV–TB in general and HIV-extrapulmonary TB in particular is significant [2, 4, 8] and enhanced efforts at early diagnosis are essential.

The Antiretroviral Therapy (ART) Centre at National Institute of TB and Respiratory Diseases (NITRD) is an HIV clinic in a referral TB Hospital providing free diagnostic and treatment services under the national TB and HIV control programs of the Government of India. With the aim to screen for pre-existing lesions and/or establish a baseline for possible future abdominal conditions (e.g. immune reconstitution inflammatory syndrome), an ultrasonography (USG) of the abdomen is carried out as part of routine pre-ART screening tests for all HIV patients entering long term care at our centre. For several years we observed USG findings suggestive of active abdominal TB in a significant number of PLHIV. Based on this observation and the results of a small prospective pilot study done to assess the role of different tests in diagnosis of HIV-extra pulmonary TB [5], we undertook this data analysis to evaluate the role of routine abdominal ultrasonography for active case finding of abdominal TB in HIV clinics.

## Methods

This study was a retrospective analysis of prospectively collected data for 1064 adult and adolescent HIV infected patients, registered between April 2006 and March 2014 at the Antiretroviral Therapy centre (ART centre) at National Institute of Tuberculosis and Respiratory Diseases, a tertiary care TB institute in New Delhi, India. All patients were ART naive at entry to HIV care. At the time of registration, HIV infected patients underwent a symptom screening, physical examination, baseline chest x-ray, sputum examination, abdominal ultrasonography (USG) and routine blood tests. Patients already diagnosed with active TB and on anti-tubercular treatment (ATT) at time of registration also underwent the pre-ART screening work up. Data in standardized case forms was available for all the cases. The case forms were scrutinized for diagnosis of TB, type of TB (pulmonary/extrapulmonary or both), site of TB if extrapulmonary TB, abdominal USG findings and CD4 cell counts. Data was extracted and entered in standard research forms. HIV cases were grouped into those already diagnosed with TB at time of ART registration and those newly diagnosed with TB at the ART Centre at the time of entry in HIV care. Abdominal USG reports were analysed in detail for the presence of findings suggestive of abdominal TB.

### Diagnosis of HIV

HIV infection was diagnosed using three antigenically different rapid kits as per the national HIV testing policy [9].

### Diagnosis of TB

Active TB in HIV positive patients already diagnosed with TB as well as those newly diagnosed at the ART Centre was as per the existing WHO and Revised National Tuberculosis Control Program (RNTCP) guidelines. The diagnosis was based on identification of typical clinical features, with isolation of acid fast bacilli (AFB) from a clinical specimen wherever possible and/or the presence of radiological (chest radiographs/USGs/CT scans) findings suggestive of TB. TB was classified as either pulmonary TB (smear-positive or smear-negative), or extrapulmonary TB or both [10].

### Diagnosis of abdominal TB

Abdominal TB in an HIV infected person was diagnosed on basis of USG (abdomen) findings with/without symptoms suggestive of TB. The USG findings and symptoms taken to be suggestive of active abdominal TB are as follows: (A) Major USG findings - i) multiple, enlarged lymph nodes in the abdomen of >1.5 cm with central necrosis seen as area of hypoechogenicity, with or without matting ii) ascites (B) Minor USG findings - i) thickened bowel loops ii) splenic microabscesses iii) hepatosplenomegaly (C) Symptoms taken to be suggestive of TB – current fever, weight loss, cough of any duration and abdominal complaints. Active abdominal TB was further classified as probable or possible. The criterion used for classifying probable and possible abdominal TB is shown in Table 1. Bacteriological confirmation of abdominal lesions was not obtained in this study; therefore no abdominal TB diagnoses were definite or microbiologically confirmed.

**Table 1:** Diagnostic criterion for probable and possible abdominal TB

Probable TB (any one of the below)	
a) Two major USG findings	
b) One major USG finding with at least two minor USG findings	
c) One major USG finding with at least two symptoms suggestive of TB	
d) Any USG finding suggestive of TB with presence of microbiologically confirmed TB at another site	
Possible TB (any one of the below)	
a) One major USG finding	
b) Two minor USG findings with at least two symptoms suggestive of TB	

### Abdominal ultrasonography

The abdominal USGs were performed on either a Samsung Medison (Model SONOACE X8) machine or a SEIMENS G50 machine, by one of two clinical radiologists (medical doctors) with more than five years experience as specialists. All USGs were reported in structured formats as a standard hospital practice.

### Data analysis

The extracted data was compiled and analysed using Microsoft Office Excel software. Continuous data is presented as median and inter-quartile range (due to extreme values) and categorical data is presented as percentages. Proportions were compared using a Fischer exact test and quantitative data was compared using a t-test. All tests were two tailed and a *p* value of <.05 was considered significant.

This study was approved by the Institutional Review Board of National Institute of Tuberculosis and Respiratory Diseases.

## Results

**Fig. 1:**
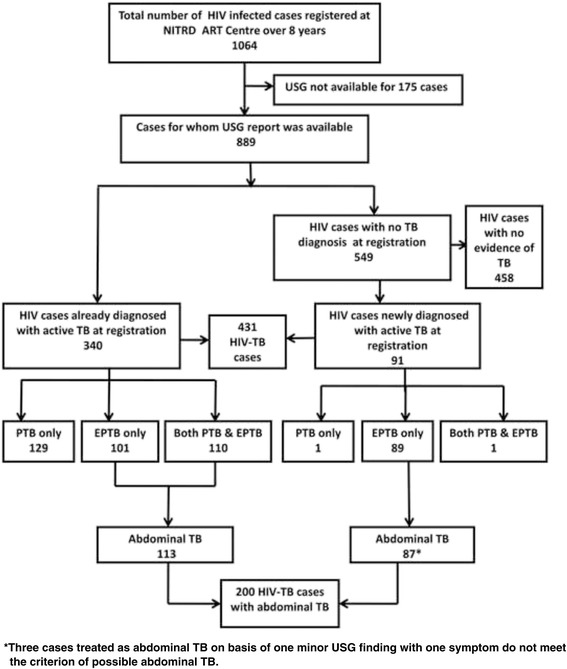
Details of HIV infected cases registered at NITRD ART Centre included in the analysis.

One thousand sixty four adult patients infected with HIV were registered at the ART Centre of National Institute of Tuberculosis and Respiratory Diseases, New Delhi between April 2006 and March 2014 for ART initiation**.** Of these, a baseline abdominal USG report was available for 889 HIV patients as part of their pre-ART screening workup (Fig. 1 shows details of HIV infected cases included in the analysis). Three hundred and forty people living with HIV were already on ATT for active TB at the time of ART registration, of which 239 had pulmonary TB (PTB) alone or in combination with extrapulmonary TB (EPTB). The remaining 101 already known cases of TB were of EPTB alone. All of the 340 cases had an USG abdomen at entry to HIV care and 113 (33.2%) subsequently revealed abdominal TB on USG abdomen, 56 of which had probable and 57 had possible abdominal TB as per our study criterion. Of the 549 HIV patients without a prior diagnosis of TB, 91 (16%) were newly diagnosed as active TB at the HIV clinic at the time of registration. These diagnoses were made as a combined result of the intensified TB case finding procedures (sputum examination and chest radiography of symptomatic cases) and due to the USG examination undertaken as a pre-ART screening test. Of these newly diagnosed TB patients, one had only PTB (sputum smear positive for AFB), one case had both PTB and EPTB (smear negative PTB with AFB positive cervical lymph node aspirate) and 89 had only EPTB. Of these 89 new TB cases with only EPTB, one case had pleural effusion and one had cervical lymphadenopathy (with cytology reports suggestive of TB). The remaining 87 cases were diagnosed as abdominal TB. Forty-one of these 87 abdominal TB patients were classified as probable and 43 as possible abdominal TB on basis of the study criterion. There were 3 patients among these 87 who did not meet the criteria of probable or possible abdominal TB but had been given ATT on the basis of unexplained fever/abdominal pain with thickened bowel loops. Their treatment may be considered as on an empirical basis. However, in all these 87 cases, the abdomen was the only site of active TB. The details of microbiologically confirmed and clinically diagnosed TB cases in both the above groups are given in Table 2.

**Table 2:** Microbiologically confirmed and clinically diagnosed HIV-TB cases

HIV cases screened	No TB or new TB	HIV-TB cases	Microbiologically confirmed TB cases	PTB +/− EPTB (microbiologically confirmed TB)	EPTB only (microbiologically confirmed TB)	Abdominal TB (Probable/ Possible)
Cases already on ATT at entry (*n* = 340)	0	340 (known TB cases)	102	239 (72)	101 (30)	113 (56/57)
Cases not known to have TB at entry (*n* = 549)	458	91 (new TB diagnosis)	2	2 (2)	89 (0)	87 (41/43) + 3 cases treated as abdominal TB empirically
TOTAL	458	431	104	241 (74)	190 (30)	200 (97/100)

*PTB=* Pulmonary TB; *EPTB=* Extra pulmonary TB; *ATT=* anti-tubercular therapy, +/− = with or without

On a whole, there were a total 458 HIV patients without TB and 431 HIV patients with active TB disease in the cohort (Patient screening characteristics are given in Table 3) and of the total 200 cases treated as abdominal TB, 197 could be classified as either probable or possible abdominal TB. Details of their abdominal ultrasonography findings are given in Table 4 and the diagnostic criterion for probable and possible TB in cases people already diagnosed with active TB and those newly diagnosed with active TB are given in Table 5. The median CD4 cell count of cases with active abdominal TB was 111 cells/μ l (IQR 68–200).

**Table 3:** Patient screening characteristics

Patient Characteristics (*N* = 889)	Only HIV cases (*n* = 458)	HIV-TB cases (*n* = 431)	*p* value
Male Gender, n (%)	296 (64.6%)	339 (78.6%)	0.0001
Media Age, years (IQR)^a^	34(30–40)	34 (30–39)	0.35
Median CD4 cell count, cells/μl (IQR)	214 (119–334)	126 (67–226)	< 0.0001

^a^Interquartile range

**Table 4:** Abdominal ultrasound examination findings in HIV cases with abdominal TB

Abdominal ultrasound examination findings	No. of patients (%)
Enlarged lymph nodes^a^, thickened bowel loops & hepatosplenomegaly	6 (3)
Enlarged lymph nodes^a^, thickened bowel loops & ascites	2 (1)
Enlarged lymph nodes^a^, splenic micro-abscesses & hepatosplenomegaly	5 (2.5)
Enlarged lymph nodes^a^, splenic micro-abscesses & ascites	2 (1)
Enlarged lymph nodes^a^, hepatosplenomegaly & ascites	1 (0.5)
Enlarged lymph nodes^a^ & thickened bowel loops	9 (4.5)
Enlarged lymph nodes^a^ & splenic micro-abscesses	16 (8)
Enlarged lymph nodes^a^ & hepatosplenomegaly	35 (17.5)
Enlarged lymph nodes^a^ & ascitis	4 (2)
Thickened bowel loops & hepatosplenomegaly^b^	12 (6)
Thickened bowel loops & ascites	1 (0.5)
Splenic micro-abscesses & hepatosplenomegaly^b^	2 (1)
Splenic micro-abscesses & ascites	1 (0.5)
Enlarged lymph nodes only	88 (44)
Ascites only	4 (2)
Thickened bowel loops only^c^	9 (4.5)
Splenic micro-abscesses only^c^	3 (1.5)

^a^Multiple abdominal lymph nodes, more than 1.5 cm in size with areas of central necrosis seen as hypoechogenecity on USG, with or without evidence of matting

^b^Two minor USG findings were supported by presence of at least two symptoms suggestive of TB or microiologically confirmed TB at another site

^c^One minor USG findings were supported by presence of microbiologically confirmed TB at another site

**Table 5:** Diagnostic criterion for probable and possible abdominal TB among cases already diagnosed and those newly diagnosed as TB at entry to HIV care

Abdominal ultrasound findings suggestive of TB^d^	Number of abdominal TB cases (*N* = 200)^a^	
	Already diagnosed with TB at another site at entry to HIV Clinic (*N* = 113^b^ / 340)	Newly diagnosed at entry to HIV clinic (*N* = 87^a,c^ / 91)
Probable abdominal TB cases (*n* = 97)		
1. Two Major findings	2	4
2. One major with at least two minor findings	1	6
3. One major finding with at least two symptoms	1	31
4. USG findings in cases with microbiolgically confirmed TB at another site:		
• Two major findings (*n* = 3) • One major & two minor findings (*n* = 4) • One major finding alone or with one minor finding/one symptom (*n* = 33) • Two minor findings with more than two symptoms (*n* = 3) • One minor finding (*n* = 9)	52	None of the abdominal TB cases in this group had TB at another site
Possible abdominal TB cases (*n* = 100)		
1. One major finding	51	38
2. Two minor findings with at least two symptoms	6	5

^a^Three cases of abdominal TB were treated on basis of unexplained fever/ abdominal pain with thickened bowel loops, however did not meet criterion of probable or possible TB

^b^Included 239 cases with pulmonary TB and 101cases with extra pulmonary TB at another site

^c^There was no pulmonary TB or extra pulmonary TB at another site these cases

^d^Major USG findings suggestive of TB – 1) Multiple abdominal lymph nodes, more than 1.5 cm in size with areas of central necrosis seen as hypoechogenecity on USG, with or without evidence of matting 2) Ascitis. Minor USG findings suggestive of TB – 1) Thickened bowel loops 2) splenic microabscesses 3) hepatosplenomegaly

Along with weight loss, fever, cough, diarrhoea and abdominal pain, the other symptoms reported were abdominal pain, breathlessness and loss of appetite (Table 6). One hundred and six (53%) patients with HIV-abdominal TB had only constitutional symptoms with no abdominal symptoms, while 54 (22%) of the 200 HIV-abdominal TB patients did not have any symptom at presentation to the ART Clinic. We found diarrhoea as the most common abdominal complaint, seen in 94 (47%) of HIV-abdominal TB patients, of which diarrhoea alone was present in 32. Cough was present in 24 of the only abdominal TB patients with no evidence of PTB.

**Table 6:** Presenting symptoms in cases with HIV-abdominal TB (*N* = 200)

Presenting symptom^a^	No. of patients (%)
Weight loss	135 (72.5)
Fever	128 (64)
Cough	96 (48)
Diarrhoea	94 (42)
Abdominal Pain	60 (30)
Breathlessness	52 (26)
Loss of appetite	45 (22.5)

^a^More than two symptoms were seen in 64.5% cases

## Discussion

Extra-pulmonary TB is a predominant form of TB in HIV infected people, of which the abdomen is a frequent site [4, 6–8, 11–13]. As abdominal TB presents with non-specific symptoms [14–16] and has a low index of suspicion, the likelihood of it’s under diagnosis becomes high. In our study too, the symptoms in the majority of HIV abdominal TB patients (53%) were constitutional or non-abdominal, that is fever, weight loss and cough. Though, these are important screening symptoms for active TB in HIV patients [17], they do not specifically indicate abdominal involvement and do not differ from symptoms seen among patients with HIV - non-abdominal TB or even HIV alone. Moreover, 32 (16%) of the abdominal TB patients had diarrhoea as the only abdominal complaint, which overlaps with the general symptoms of HIV and 22% reported no symptoms at all. This implies that it would be easy to miss the diagnosis of abdominal TB in clinical practice, unless specifically looked for, especially if general TB symptoms are associated with a negative chest radiograph and sputum examination result.

In our study, 79% (*n* = 340) of the HIV-TB patients had a prior diagnosis of TB at entry to HIV care. An USG in these cases showed features suggestive of abdominal TB in a high proportion (33.2%) of cases. This is mainly due to the fact that this group was a preselected population of TB cases and abdomen was an additional site of TB and a manifestation of dissemination of TB in the context of HIV [8, 17]. While in HIV patients with no prior diagnosis of TB (*n* = 549), a much lower proportion of abdominal TB was diagnosed (15.8%, *n* = 87). Nevertheless, this too is a significant number of TB cases diagnosed and in this group of patients the role of a routine USG is especially relevant for timely diagnosis and treatment. In cases already diagnosed with TB at entry to HIV care, abdominal USG may have limited usefulness as these patients will in any case receive TB treatment, treating abdominal TB as well.

Though a microbiological diagnosis is difficult to obtain for abdominal TB, a simple ultrasonic examination of the abdomen, which is both easy to perform and widely available, is mostly sufficient to diagnose abdominal TB. Unlike the symptoms, there are some typical (and multiple) radiological features suggestive of tubercular disease activity [18, 19]. Enlargement of multiple abdominal lymph nodes (> 1.5 cm), with or without matting, with zones of hypoechogenecity representing caseation, hypoechoic lesions in the spleen indicative of splenic microabscesses, bowel loop thickening and ascites are the main features considered suggestive of active TB in the abdomen by several authors of HIV-TB studies, from India and globally [4, 20–24]. In our patients, enlarged abdominal lymph node with central necrosis, a finding which is easy to detect on USG abdomen, requiring minimal training and a simple USG machine, was the common USG finding. CT scan confirmation is necessary in few cases [5]. Interestingly, only 4 of our HIV-abdominal TB patients, had ascites alone, and ascitis with other USG findings was seen in 9 more cases. This lower number of ascitis cases may be due to the fact that ascites is easier to diagnose clinically and these cases might have been diagnosed earlier (in gastroenterology or general surgical clinics) without the need for active case finding. Moreover, ascites in some cases may have rapidly cleared with treatment, therefore not see on USGs done after a certain duration of anti-tubercular therapy [25]. There is some evidence to suggest that TB ascites is less frequent in HIV infected as compared to HIV uninfected case [2], though a study done by Sinkala et al. at a referral hospital in Zambia, reports ascites on USG in a high proportion of abdominal TB-HIV cases (16/22) [24]. The reason for higher number of ascites cases in this study maybe due to the study population of hospitalized symptomatic HIV patients, in whom ascites was one of their criteria for patient inclusion to further undergo a laparoscopy / colonoscopy to obtain a clinical sample for microbiological examination. Their findings may be expected to differ from this study which is done in an out-patient clinic and included asymptomatic cases.

The differential diagnosis for HIV infected people with abdominal USG findings as seen in this study (enlarged lymph nodes, hepatosplenomegaly, bowel loop thickening), especially considering the low CD4 cell count of our cases, would mainly be disseminated non-tuberculous mycobacteria- *Mycobacterium avium complex* (MAC), fungal diseases like histoplasmosis, gram negative enteric infections like salmonellosis, Kaposi’s sarcoma and lymphomas [26]. Diagnosis of abdominal lesions is usually made simpler in these conditions by coincident involvement of other sites like lung, skin or peripheral lymph nodes. Additionally, mycobacterial blood cultures for MAC, fungal blood cultures, antigen detection tests for *Histoplasma* andstool/blood cultures for *Salmonella* and other bacterial infections would be useful in arriving at a diagnosis [27, 28]. However, in the case of Kaposi’s sarcoma and lymphomas, where no peripheral lesions are present, a diagnostic laparoscopy would be required for tissue biopsy and histopathological examination [29].

In this paper we highlight the problem of diagnosing abdominal TB in people living with HIV. Despite the frequency of the problem and availability of a simple and easy method of diagnosis, abdominal TB is under diagnosed in people living with HIV as there is no specific presenting symptom or sign [2, 3, 14–16]. As per current intensified case finding (ICF) protocols, HIV infected persons first go through symptom screening and those found to be symptomatic undergo a sputum examination and chest radiograph as frontline tests. An abdominal USG would usually not be ordered by the physician for an HIV-TB presumptive in the absence of abdominal symptoms. At our Centre, in addition to following the ICF protocol, we undertake USG (abdomen) as part of pre-ART screening investigations for all cases (with or without known TB) and were able to identify 200 cases of abdominal TB on the basis of the USG findings, in 87of which the abdomen was the only site of active TB. Given the non specificity of symptoms of abdominal TB, in some of these HIV cases the diagnosis of active TB might have been missed or delayed in the absence of a USG abdomen, despite routine ICF.

Our study is limited by the absence of microbiological (smear/culture) confirmation of abdominal TB, which precludes the availability of drug sensitivity testing (DST). Mycobacterial drug sensitivity testing, an important laboratory investigation in TB, is especially critical for deciding further treatment options in patients not responding to TB treatment. The absence of DST is a serious limitation of USG based diagnosis of abdominal TB. Moreover, this study was conducted in a referral TB hospital, due to which a majority of the HIV patients who presented were already diagnosed with active TB at the time of referral to HIV care. Therefore results might vary if only general HIV infected populations are screened. For HIV patients already diagnosed with active TB at the time of registration to the HIV clinic, detailed information on TB diagnosis and treatment (e.g. prior duration of TB treatment etc) were not available, correspondingly we could not analyze the relation of USG findings and symptoms to these parameters. Also, we could not analyse the effect of varying ranges of CD4 counts on the presentations of abdominal TB as the CD4 counts of our patients were quite low on a whole. Nonetheless, it is a precise retrospective review of data from a national TB Institute which cares for TB and HIV patients referred from all over north India.

## Conclusions

Abdominal TB is a major site of active TB in HIV infected cases accessing HIV care in a free HIV Clinic in North India, and at times it may be the only TB site. A majority of HIV patients with abdominal TB presented either with non-specific or no symptoms, due to which TB may go unsuspected. An abdominal ultrasonography is a simple, easily available and specific modality for diagnosis of abdominal TB. An abdominal ultrasound needs to be part of intensified TB case finding algorithms, alongside sputum examination and chest radiographs, at HIV clinics in high TB burden countries to allow improved active TB detection and subsequent timely treatment.

## Abbreviations

AFB: Acid fast bacilli
ART Centre: Anti Retroviral Therapy Centre
ART: Antiretroviral therapy
ATT: Anti tubercular therapy
DST: Drug sensitivity testing
EPTB: Extra pulmonary tuberculosis
HIV: Human immunodeficiency virus
ICF: Intensified case finding
IQR: Interquartile range
MAC: *Mycobacterium avium intercellulare*
NITRD: National Institute of TB & Respiratory Diseases, New Delhi
PLHIV: People living with HIV
PTB: Pulmonary tuberculosis
RNTCP: Revised National TB Control Program
TB: Tuberculosis
**USG**: Ultrasonography
WHO: World Health Organization
